# Hemispheric Patterns of Recruitment of Object Processing Regions in Early Alzheimer’s Disease: A Study Along the Entire Ventral Stream

**DOI:** 10.3233/JAD-220055

**Published:** 2023-01-31

**Authors:** Nádia S. Canário, Lília P. Jorge, Isabel J. Santana, Miguel S. Castelo-Branco

**Affiliations:** a Coimbra Institute for Biomedical Imaging and Translational Research, University of Coimbra, Coimbra, Portugal; b Institute for Nuclear Sciences Applied to Health, University of Coimbra, Coimbra, Portugal; c Department of Neurology, Centro Hospitalar e Universitário de Coimbra, Coimbra, Portugal; d Faculty of Medicine, University of Coimbra, Coimbra, Portugal; e Center for Innovative Biomedicine and Biotechnology, University of Coimbra, Coimbra, Portugal

**Keywords:** Alzheimer’s disease, fMRI, recognition, ventral visual stream

## Abstract

**Background:**

Investigation of neural response patterns along the entire network of functionally defined object recognition ventral stream regions in Alzheimer’s disease (AD) is surprisingly lacking.

**Objective:**

We aimed to investigate putative functional reorganization along a wide-ranging network of known regions in the ventral visual stream in mild AD.

**Methods:**

Overall we investigated 6 regions of interest (5 of which were not investigated before), in 19 AD patients and 19 controls, in both hemispheres along the ventral visual stream: Fusiform Face Area, Fusiform Body Area, Extrastriate Body Area, Lateral Occipital Cortex, Parahippocampal Place Area, and Visual Word Form Area, while assessing object recognition performance.

**Results:**

We found group differences in dprime measures for all object categories, corroborating generalized deficits in object recognition. Concerning neural responses, we found region dependent group differences respecting a priori expected Hemispheric asymmetries. Patients showed significantly decreased BOLD responses in the right hemisphere-biased Fusiform Body Area, and lower left hemisphere responses in the Visual Word Form Area (with *a priori* known left hemispheric bias), consistent with deficits in body shape and word/pseudoword processing deficits. This hemispheric dominance related effects were preserved when controlling for performance differences. Whole brain analysis during the recognition task showed enhanced activity in AD group of left dorsolateral prefrontal cortex, left cingulate gyrus, and in the posterior cingulate cortex— a hotspot of amyloid-β accumulation.

**Conclusion:**

These findings demonstrate region dependent respecting hemispheric dominance patterns activation changes in independently localized selective regions in mild AD, accompanied by putative compensatory activity of frontal and cingular networks.

## INTRODUCTION

Alzheimer’s disease (AD) is the most prevalent age-related neurodegenerative disease leading to severe cognitive impairment and affecting the capacity to function independently [1, 2]. AD progression can be subdivided into different stages with cumulative cognitive deficits that go beyond the known memory impairment, which is present early in the course of the disease [3, 4]. Several studies suggest, that contrary to impairment in memory, orientation and abstract reasoning [5–8], visual operations like the processing of shapes [9–11] may not be significantly affected in early stages. Evidence of impairment in visual recognition tasks are in most cases attributed to impairment in semantic rather than perceptual processing [10, 12–16].

Previous functional neuroimaging studies addressing visual perception and recognition, in either mild AD or mild cognitive impairment (MCI), suggested relatively preserved ventral visual stream functions [17], contrasting with evidence pointing to an impairment in the dorsal visual stream function and/or dorsoventral integration [17–22]. Early PET studies had also suggested abnormalities in the dorsal pathway in this condition [23, 24]. The dorsal and ventral pathways are two processing streams related to vision for action and object recognition, respectively [25–27]. Parvocellular channels in V1 convey information from the retina to V4 and to parts of the inferior temporal cortex involved in shape and color processing, with a small contribution of magnocellular channels [26]. In turn, the dorsal visual pathway, responsible for spatial encoding and motion processing [25–27], receives visual information mainly through magnocellular channels which project information to V3a, V5, to the middle temporal area, medial superior temporal area, posterior parietal lobule, and to intraparietal regions[25, 27].

There have been several attempts to study visual processing and recognition in AD using neuroimaging techniques. For instance, one study, using a functional magnetic resonance imaging (fMRI) paradigm with location (evoking dorsal pathway recruitment) and face perception (involving the ventral pathway responses) processing demands, suggested that the magnocellular pathway is more affected by the neurodegenerative processes occurring in amnestic MCI (aMCI) [17], being in congruence with subsequent studies investigating the dorsal stream pathway in AD-type neurodegeneration [18–21, 28, 29]. As far as we know, only few other studies explored the functional response profiles of the ventral visual stream in clinical or preclinical AD [19, 30]. These studies focused mainly on fusiform face area (FFA) and superior temporal sulcus (STS), which belong to the core face recognition network. One study investigated FFA and STS responses, in MCI patients, finding a surprisingly larger activation in FFA site for scrambled faces (in which low level abstract features dominate) in the MCI group when compared to more ecological face material [19]. However, it is worth mentioning that the recognition of faces in their work was provided by structure from motion cues, also requiring the recruitment of the dorsal visual stream, featuring dorsoventral integration. Other authors found no differences for the FFA BOLD activity with more conventional stimuli when AD patients were compared with controls [30].

The nature of visual deficits in early AD and the relative role of dorsal and ventral streams remain under discussion [19]. Thus, given the functional relevance of investigating the entire network of visual recognition areas in AD this study aims to investigate functional patterns of response in a large set of areas of the ventral visual stream in mild AD. As far as we know, no other study as yet performed a such comprehensive study on visual recognition areas in this disease. Some ventral regions involved in object recognition were never investigated, such as the fusiform body area (FBA) and the extrastriate body area (EBA) for processing of body images, the ventral part of the lateral occipital cortex (LOCv) for general object recognition, the parahippocampal place area (PPA) for the recognition of scenes, and the visual word forma area (VWFA) for the recognition of verbal material. We also explored the FFA for face image recognition.

Taking into account the *a priori* known functional specialization of the areas under study, as well as their hemispheric lateralization pattern, we expected to find regional between-group differences in the whole ventral visual stream pathway. We are also expecting for the 1-back task to impose more cognitive demands to AD participants which might be reflected in different whole brain activation patterns between groups, possibly reflecting either activation loss or compensatory mechanisms.

With this work, we expect to provide a comprehensive perspective on whether mild AD affects the function of independently identified ventral visual stream areas, implicated in higher order visual perception and recognition.

## MATERIALS AND METHODS

### Participants

A total of nineteen patients with the diagnosis of mild AD were enrolled in the study, between 2017 and 2019. All patients had a recent diagnosis of AD (less than 2 years), being at a mild stage of dementia according to the Clinical Dementia Rating (CDR = 1). All patients were recruited at the Neurology department of the Centro Hospitalar e Universitário de Coimbra (CHUC) and underwent a comprehensive neuropsychological assessment at the hospital facilities. The diagnosis of AD was performed by two experienced neurologists (including IS) at the Memory Clinic of the Neurology department of CHUC, according to results of standard clinical evaluation, cognitive assessment, laboratory tests, imaging, and Apolipoprotein E allele genotyping. For this particular study, we only considered patients with a diagnosis of AD supported by biological biomarkers (cerebrospinal fluid (CSF) or PiB-PET). The neuropsychological evaluation comprised cognitive instruments including the Mini-Mental State Examination with Portuguese normative data [31, 32], the Montreal Cognitive Assessment (MoCA) [33–35], and a comprehensive neuropsychological battery with normative data for the Portuguese population –Bateria de Lisboa para Avaliação de Demências [36], exploring memory and other cognitive domains. MRI contribution for AD diagnosis focused on exploring hippocampal and/or posterior parieto-temporal atrophy, using visual rating scores. For CSF biomarkers, pre-analytical and analytical procedures were done in accordance with previously proposed protocols [37] and all measurements were controlled for external quality according to the scheme of the Alzheimer’s Association Quality Control Program for CSF Biomarkers [38]. Patients were positive for both Aβ and tau (A + /T+). The cut-off values used in our laboratory for clinical practice have been reported before [39, 40]. AD criteria were based on the Diagnostic and Statistical Manual of Mental Disorders –fourth edition (DSM-IVTR) [41] and the National Institute on Aging-Alzheimer’s Association workgroups on diagnostic guidelines for AD [42]. All patients were in a stable condition, did not sustain recent changes in medication, and did not have ophthalmological or neurological/psychiatric conditions other than AD.

The control group was composed of nineteen age (t (36)=0.024, *p* > 0.980), gender (*p* > 0.999) and education-matched (*U* = 127, *p* > 0.111) participants. All participants from the control group were individuals from the community with a) no history of central nervous system disorders; b) no mental diseases; c) no severe sensory impairment, especially visual and/or auditory; d) were not taking medication; and e) did not sustain any other condition that could preclude the fMRI study. Participants from the control group underwent a brief cognitive assessment in order to screen for the presence of cognitive impairment, meaning that control participants did not sustain significant memory complaints, as assessed by the Subjective Memory Complaints questionnaire (SMC, mean±sd, 1.78±1.52) [43], had a normal general cognitive function according to Montreal Cognitive Assessment [35], had preserved daily living activities measured by Lawton & Brody scale (L&B, for female = 8 (constant)/for male = 5 (constant)) [44, 45] and had no indication of either moderate or severe depressive symptoms according to the Geriatric Depressive Scale (GDS-30, mean±sd 6.72±6.15) [46, 47]. Exclusions based on possible cognitive impairment included the presence of a diminished general cognitive status, which may or may not be accompanied by the presence of subjective memory complaints. Rejections based on MoCA scores were set to be more than 2 standard deviations (SD) below the respective mean [35]. Portuguese data for the MoCA provide mean and standard deviations for different age and educational level ergo we compared each participant’s MoCA score with the score that is considered to be normal according to these features. Table 1 summarizes information regarding age, education, gender distribution, and MoCA scores for both AD and controls.

**Table 1:** Demographic characteristics and performance on the Montreal Cognitive Assessment (MoCA) for both AD and controls. Mini-Mental State Examination (MMSE) scores are also depicted for the AD group

	Alzheimer’s disease	Controls
	(*n* = 19)	(*n* = 19)
Age (mean±sd)	66.11±7.02	66.05±6.77
Education (mean±sd)	8.95±5.83	10.53±5.31
Gender (m: f)	10: 9	10: 9
MoCA (mean±sd)	14.26±4.31	24.94±3.62
MMSE (mean±sd)	23.1±2.97	–

All participants provided written informed consent. The present study complied with the Declaration of Helsinki and was approved by the Ethics Committee of the Faculty of Medicine, University of Coimbra.

### Stimuli and procedures

**Fig. 1:**
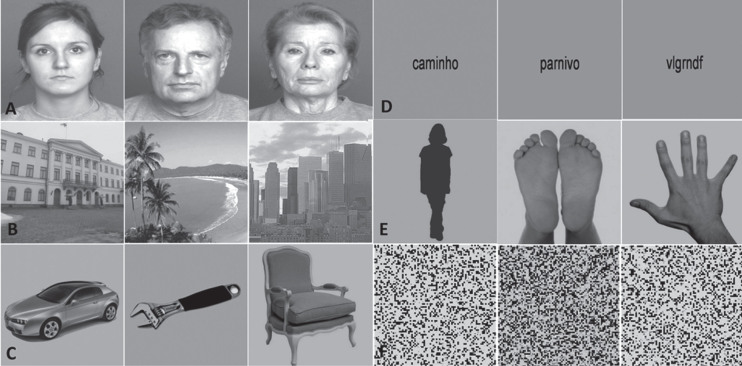
Example of the images presented in the n-back functional task. Left to right: A) young, middle, and old faces; B) buildings, landscapes, and skylines; C) cars, tools, and chairs; D) words, pseudowords, and nonwords; E) body shape silhouettes and hands & feet; F) scrambled stimuli.

All stimuli presented during this experiment are described elsewhere [48, 49]. Overall, 6 different types of grey-scale visual stimuli were delivered to all the participants included in the study. The set of stimuli comprised images of faces, bodies, objects, places, verbal material and scrambled images, and each one of these categories were divided in three different subcategories of objects. Thus, the face category was composed by faces of young, middle age and old persons. The bodies category comprised faceless body stimuli, images of hands and feet (hands&feet) and body shape silhouettes. For the object category, we presented tools, cars, and chairs, whereas the place category comprised landscapes, buildings, and skylines. Verbal stimuli were composed by words, pseudowords, and nonwords. Scrambled stimuli consisted of images with no semantic meaning, which included unrecognizable images from all the other categories. All face stimuli were taken from the FACES database [50]. Faceless body images were taken from Bochum Emotional Stimulus Set database [51], whereas images of hands and feet were selected from publicly available online images and body shape silhouettes were created using a customized code in MATLAB R2014a (MathWorks, Natick, USA). Like the hands & feet subcategory, all stimuli from the objects’ category were taken from publicly available online images, and all images from the places’ category were taken from the database of the computational visual cognition laboratory [52] (http://cvcl.mit.edu/database.htm). Verbal material was provided as a courtesy from the database of Universidade Católica Portuguesa. Lastly, scrambled images were created using a custom written algorithm in MATLAB, which divides each intact image into a grid of size 50 x 50 and 40 x 40, with the tiles being randomly shuffled, and filling 0.22° and 0.28° of the visual field, respectively. All stimuli were grey-scale images and were equalized for luminance and contrast with SHINE toolbox [53] (for additional details, see [48] and [49]. Examples of the visual categories used in this experiment are depicted in Fig. 1.

This experiment comprised two functional block-design runs in which the stimuli were randomly presented. Each one of the runs comprised 18 pseudo-randomized blocks covering all the subcategories— 3 blocks for each stimulus category. Each block was composed of 20 images, belonging to a particular subcategory, each one presented for ∼800 ms followed by a ∼200 ms of interstimuli interval which represents an individual duration of 20 s per block. Each block was separated by 10-s fixation with uniform grey-scale image baseline interval, which was delivered as the baseline condition. Subjects were instructed to press a button every time the image being presented was the same that had been presented immediately before (1 back-task). Each block had always four repetitions of images, that is, four possible targets to which participants had to respond to. This means that participants had a total of 12 chances for hits per stimulus category in each run. Before the scan session, subjects performed a brief training session (∼60 s) in order to guarantee that they understood the task demands and to be familiarized with the stimuli. All stimuli presented during the functional runs were delivered by a computer onto an LCD screen with 1920*1080 resolution at the head of the scanner with an angled mirror positioned on the head-coil, and were presented using Presentation 17.1 software (Neurobehavioral systems). The images’ size used to build the stimuli was 544 x 544 pixels and subtended approximately 11° x 11° of visual field. Behavioral responses were collected during acquisition via a response box and stored in a log file.

### fMRI acquisition

Data acquisition was performed in a 3 Tesla Siemens Magneton Trio scanner with a 12-channel head matrix coil. Each session started with a T1-weighted 3D anatomical MPRAGE (rapid gradient-echo) sequence, using a voxel resolution of 1.0 x 1.0 x 1.0 mm. Repetition time (TR) was set at 2530 ms, echo time (TE) at 3.42 and had a field of view (FOV) of 256 x 256 mm. Each MPRAGE sequence comprised 176 slices, a flip angle of 7º and an inversion time of 1100 ms. T2*-weighted 2D echo-planar images were obtained in 2 functional runs, lasting for ∼9 min. Acquisition parameters for the functional runs were: TR = 2000 ms, TE = 30 ms, voxel size = 2.5 x 2.5 x 3 mm, FOV = 256 x 256 mm, matrix size = 102 x 102 and a flip angle of 90°. Each functional localizer sequence comprised 31 slices and had 276 volumes.

### Pre-processing and statistical analysis

Anatomical images were corrected for the inhomogeneity of signal intensity, re-oriented into AC-PC plane and further transformed to the Talairach reference system. fMRI data were slice scan time-corrected, temporal-filtered, corrected for signal intensity, and motion-corrected. The pre-processed fMRI in fMRI native space was coregistered with the anatomical scan also in MRI native space. The resulting image was then transformed to the Talairach space using the transformation between MRI native space and Tailarach space determined previously using the anatomical data. The voxel size for the resampled fMRI data in Talairach space was 1.0 x 1.0 x 1.0 mm^3^. All the steps above are described in more detail elsewhere (see [54]).

We localized, at a group level, based on independent contrast-based localizer approaches using data from both AD and control groups, and known anatomical locations, a total of 6 regions of interest (ROIs) in both hemispheres belonging to the ventral visual stream: FFA, FBA, EBA, LOCv, PPA, and VWFA. For that we performed a multi-subject random-effect general linear model (RFX-GLM) analysis, which allows to calculate the estimated effects (beta values) for each functional predictor using each subject specific time course. This multi-subject design matrix allows the results to be generalized to the population since it simulates the inter-subject variability in the data. After ROI definition we have checked the data for significant inter-subject variability, where our data showed similar standard deviations between groups for all ROIs. Furthermore, and as a sanity check, we also ran a GLM for the AD group alone and replicated the identification all the 7 regions of the ventral visual stream under study. This procedure was taken as a control measure in order to check if the previous group ROIs identified using the time course of both groups includes the time course of the AD groups with no significant shifting of boundaries occurring for the latter group.

Our analysis plan was to investigate for group differences across the independently identified brain regions, taking into account their hemispheric dominance.

First, we aimed at independent region identification also based on known anatomical locations. Statistical t maps for each region of interest were obtained using the following functional contrasts according to the functional specificity of each region and both groups: for the FFA we used the conjunction contrast [faces > places] AND [faces > scrambled] AND [faces > objects], for the FBA and EBA we used [bodies > objects], for the LOCv we used [bodies > scrambled] and for the VWFA we selected [verbal > scrambled]. We have also used a conjunction contrast in order to identify the PPA –AND [places > objects]. All contrasts were previously reported in functional mapping studies of visual recognition regions in healthy participants [48, 49] and were set according to the functional specificity of the regions.

Statistical thresholds for each t-map were set independently to localize each ROI (given that their detectability is distinct), with the least conservative threshold set on 0.01 uncorrected (just for localization purposes). For the areas found only in one hemisphere (because of high hemispheric lateralization), we used the principle of homotopy [55, 56] to define a mirror ROI using the same anatomical features (i.e. number of voxels and their respective coordinates) as the first. This procedure was done for the right VWFA, and similar to functional definition of ROIs, it was also performed at the group level, so that post hocs conducted using each participant estimated effects came from the same objectively defined homotopic ROIs. The use of mirror ROIs was done in order to perform unbiased comparisons between the ROIs and is a valid procedure previously used in earlier studies [55, 56].

After independent identification of ROIs, we proceeded to the main analysis of interest. The localizer and analysis contrasts are different because the first are subtractive, focusing on specificity, and the latter does not rely on any subtractive contrast. In this step, we resorted to betas from ROI’s preferred category, that is, for the FBA we extracted the betas from bodies stimuli, and for the VWFA we used the betas from verbal stimuli. The same criteria were used for the remaining ROIs. Each value extracted for the estimated effect was corrected for serial correlations.

Given our *a priori* hypothesis we performed direct regional between-groups comparisons for each ROI taking into account known hemispheric dominance and regionally preferred object category. Thus, for the FFA we contrasted the betas from faces between groups in order to test whether FFA’s known sensitivity to faces is affected in AD; for the FBA and EBA, we contrasted the betas from bodies in order to test whether FBA and EBA’s sensitivity to bodies is affected in AD, as well as for the other regions.

In addition to the ROI-based analysis, we also investigated whole-brain differences between groups in order to explore BOLD differences in the most difficult task condition. For that we performed a multisubject RFX-GLM analysis using the contrast [scrambled > baseline]. The scrambled was the predictor that had lower dprime in the 1-back task (see behavioral results above). Statistical t maps were obtained with a threshold < 0.01 corrected using the cluster threshold estimator (1000 montecarlo simulations).

Both pre-processing of functional data and the RFX-GLM procedures were computed in Brainvoyager QX 2.8.2 (BrainInnovation, Maastricht, the Netherlands).

For the behavioral analysis, main dependent behavioral variables were dprime, omissions, and the reaction times (RT). Dprime (d’) is a sensitivity measure that corrects for the response criteria, and are computed by subtracting the normalized hit rate (Z_Hit_) to the normalized false-alarm (FA) rate (Z_FA_), using the following formula d’=Z_Hit_ - Z_FA . _. Behavioral results were explored using the Mann-Whitney test.

Post-hoc analyses were corrected for multiple comparisons either using the Bonferroni or the Benjamini-Hochberg method (*q* = 0.1). Statistical significance was determined as < 0.05 two-tailed level.

Both behavioral and further analysis on the estimated effects from the fMRI data, were performed on IBM SPSS statistical package (v.24)

## RESULTS

### fMRI results: ROI-based approach

**Fig. 2:**
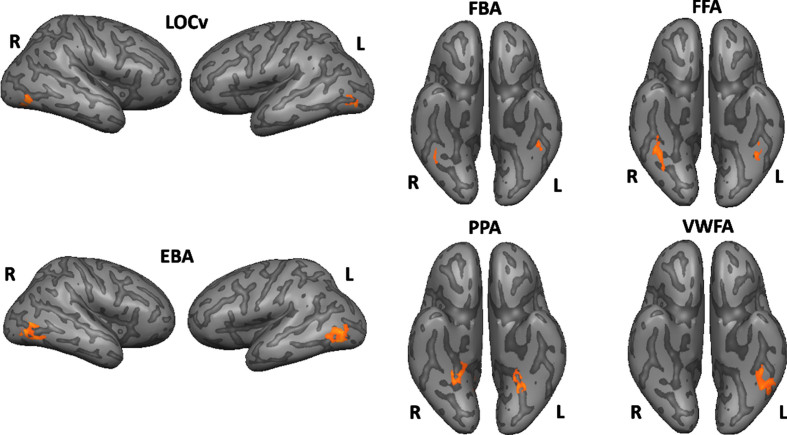
Functionally defined ROIs using subject-specific volume time courses (the surface maps are only for visualization purposes).

**Fig. 3:**
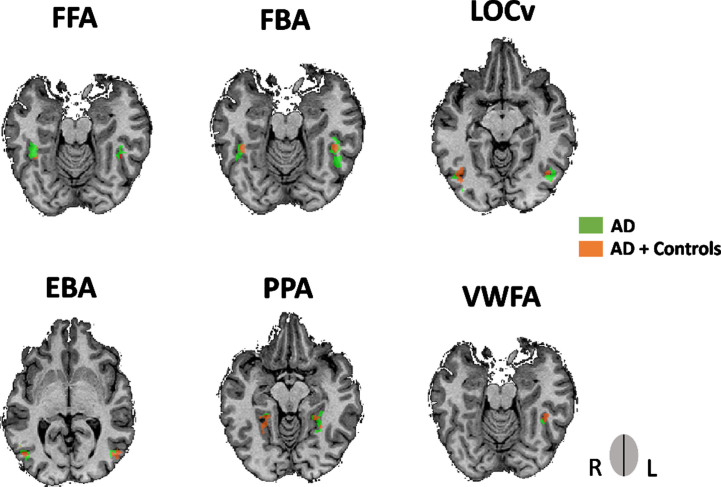
ROIs obtained from the multisubject GLM for the AD group showing an overlap with the ROIs obtained from both groups (AD and controls). The former ROIs was only performed as a control measure. Legend: rFFA, AD’s PV = 39, -40, -17; lFFA, AD’s PV = -42, -43, -20; rFBA, AD’s PV = 39, -43, -17; lFBA, AD’s PV = -45, -43, -14; rEBA, AD’s PV = 45, -73, 1; rEBA, AD’s PV = -48, -67, 7; rLOCv, AD’s PV = 45, -71, -8; lLOCv, AD’s PV = -42, -67, -8; rPPA, AD’s PV = 24, -40, -8; lPPA, AD’s PV = -27, -46, -11; lVWFA, AD’s PV = -39, -46, -17.

All ROIs identified are depicted in Fig. 2. For more details, see Table 2. See also Fig. 3 to view all regions identified in each group separately (control procedure).

**Table 2:** Talairach coordinates for the peak voxel and center of mass, maximum t value for the specified contrast and the number of voxels

ROIs		Peak Voxel	Center of mass	Max. t	No. voxels
FFA	*Right*	30, -41, -18	39, -43, -19	6.030	622
	*Left*	-40, -41, -18	-40, -44, -19	4.490	221
FBA	*Right*	41, -41, -15	40, -43, -16	4.320	192
	*Left*	-43, -41, -15	-43, -42, -17	4.703	267
EBA	*Right*	44, -77, -7	46, -70, -3	5.992	263
	*Left*	-39, -68, 6	-48, -70, 3	6.704	651
LOCv	*Right*	44, -68, -12	41, -69, -9	7.962	560
	*Left*	-43, -65, -9	-43, -68, -11	6.703	293
PPA	*Right*	26, -50, -9	24, -44, -11	6.764	342
	*Left*	-28, -50, -12	-26, -46, -11	6.875	207
VWFA	Right*	39, -41, -15	-41, -42, -18	2.294	266
	*Left*	-40, -44, -18	-41, -42, -18	6.439	266

*Depicts the mirror (homotopic) ROI.

Direct comparisons between groups revealed and using ROIs’ preferred category (i.e., bodies for FBA, verbal for VWFA, for example) that the control group had higher estimated effects for both the FBA and the VWFA compared to AD patients in the preferred hemisphere (Right hemisphere: controls > AD for FBA, t (36) = 2.957, FDR corrected, / Left hemisphere: controls > AD for the VWFA (t (36) = 2.918, FDR corrected). No differences between groups were found for the other ROIs. See also Supplementary Tables 1 and 2.

Since the AD group showed worse performance on the behavioral measures, we further investigated if the differences found for the right FBA and the left VWFA could be explained by the performance on the task for the respective preferred stimuli of each area. Single-factor ANCOVA for both right FBA and left VWFA still showed a Group effect (FBA: F (1) = 5.798, *p* < 0.022; VWFA: (F (1) = 3.331, *p* < 0.021).

### fMRI results: whole-brain approach

**Fig. 4:**
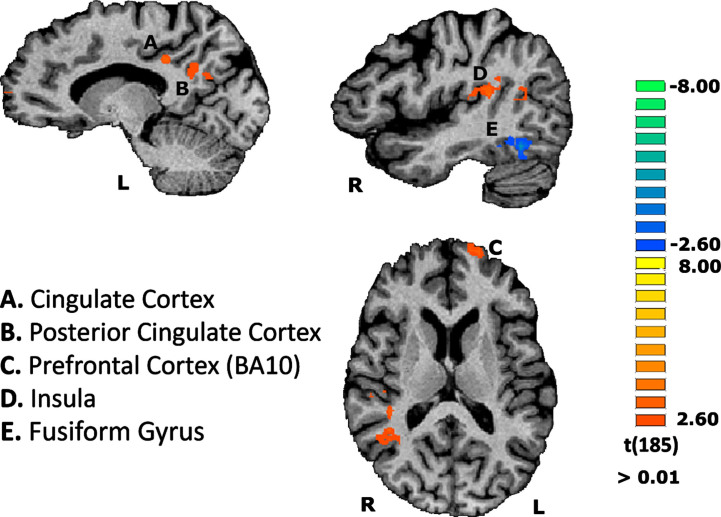
Group differences concerning the contrast of scrambled stimuli vs baseline, on the functional task. Results are depicted showing regions differentially activated in AD compared to controls. Region A: CM = -15, -37, 34; PV = -13, -35, 33; Nr. Voxels = 330 / Region B: CM = -11, -56, 26; PV = -10, -56, 30; Nr. Voxels = 649 / Region C: CM = -18, 63, 11; PV = -19, 64, 12; Nr. Voxels = 329 / Region D: CM = 42, -32, 18; PV = 47, -23, 18; Nr. Voxels = 989.

This RFX-GLM subsequent exploratory analysis investigated whether there is a distinct pattern of areas when both AD patients and controls tried to recognize the most difficult stimuli. This aimed to explore possible compensatory brain activation in regions dedicated to deal with cognitive effort for the AD group. Indeed, the former group, compared to the controls, revealed significant activations in specific brain areas. These regions mainly included the left dorsolateral prefrontal cortex (DLPFC)— ∼BA10— and left cingulate gyrus. We also found activations in posterior cingulate cortex (PCC) and in right posterior insula in the AD group in comparison to the control group (see Fig. 4).

### Behavioral results

Mean and respective SD for dprime and RT are depicted in Table 3. We found differences between groups in both dprime and omissions. Differences in dprime were found for all stimuli (dprime: objects, *U* = 62.50, p_corrected_ < 0.004; faces, *U* = 45.50 p_corrected_ < 0.001; bodies, *U* = 40.50, p_corrected_ < 0.001; places, *U* = 87.00 p_corrected_ < 0.037; verbal, *U* = 68.00, p_corrected_ < 0.007; scrambled, *U* = 54.00 p_corrected_ < 0.002 / omissions: objects, *U* = 79.50, p_corrected_ < 0.017; faces, *U* = 71.50, p_corrected_ < 0.009; bodies, *U* = 63.00, p_corrected_ < 0.004; places, *U* = 91.50 p_corrected_ < 0.06; verbal, *U* = 73.00, p_corrected_ < 0.010; scrambled, *U* = 119.00, *p* > 0.071). No differences between groups were found for the RT (objects, *U* = 158.00 *p* > 0.510; faces, *U* = 148.00 *p* > 0.342; bodies, *U* = 153.00 *p* > 0.421; places, *U* = 148.00 *p* > 0.342; verbal, *U* = 167.00 *p* > 0.692; scrambled, *U* = 142.00 *p* > 0.716).

**Table 3:** Mean and s.d for dprime, omissions, and RT found for both AD and controls on the functional task

	Alzheimer’s disease			Controls		
	Dprime	Omissions	RT	Dprime	Omissions	RT
	(*m*±sd)	(*m*±sd)	(*m*±sd)	(*m*±sd)	(*m*±sd)	(*m*±sd)
Faces	1.71 ± 1.17	1.55 ± 1.00	720.47 ± 247.35	4.36 ± 2.44	0.57 ± 0.58	691.95 ± 84.49
Objects	1.58 ± 1.88	1.77 ± 1.24	642.68 ± 188.06	3.41 ± 1.94	0.67 ± 0.73	663.34 ± 84.79
Scrambled	-0.08 ± 1.63	2.47 ± 0.95	650.96 ± 226.08	1.95 ± 1.46	1.82 ± 1.06	643.08 ± 95.85
Bodies	1.05 ± 1.02	1.97 ± 1.09	679.49 ± 392.22	3.45 ± 2.06	0.74 ± 0.85	681.93 ± 102.40
Places	2.13 ± 2.42	1.74 ± 1.33	613.50 ± 191.36	4.03 ± 2.28	0.68 ± 0.92	668.16 ± 90.98
Verbal	1.83 ± 1.84	1.87 ± 1.32	699.92 ± 175.82	4.23 ± 2.45	0.66 ± 0.86	699.27 ± 91.00

## DISCUSSION

In this study, we investigated functional neuronal changes across the entire ventral visual pathway in mild AD, by analyzing for the first time the functional response of a wide range of independently defined visual object recognition areas. It remains a matter of debate if higher order visual processing along the ventral visual stream is affected in AD [2, 5, 57]. Explicit mapping of visual object recognition regions along this pathway, as done here, is critical to answer this question.

Our results reveal region dependent group differences that provide a novel insight on the controversy concerning higher order visual processing in mild stages of AD. The idea that at least some parts of the ventral visual pathway might be relatively spared in early or pre-AD are in accordance with previous functional studies. One study in aMCI, failed to find group differences on the face selective region during a face matching task [17]. However, enhanced activation in frontal lobe regions was found for the spatial location task (eliciting activation within the dorsal visual pathway), which was interpreted as a compensatory mechanism [17]. Another study [30] using a face categorization task, found no differences in FFA’s response between AD and controls after controlling for accuracy. In the study by Bokde et. al [17], there were no differences in activation between MCI and control group in both ventral and dorsal visual pathways, during a face matching task. Moreover, no between group differences in the ROI analysis of fusiform regions were found. Although the results were described based on the assumption that their clinical sample were at high risk for developing AD, authors analyzed the functional response of amnesic and multiple domain MCI (aMCI; mdMCI, respectively) and not clinically probable AD patients. There is indeed a link between aMCI (especially isolated aMCI) and AD as about 80% develop AD [58, 59], but testing a heterogeneous group of people with aMCI without support by AD biomarkers [60] may further explain their negative results. Interestingly, our own previous aMCI fMRI study on perception of structure-from motion 3D faces showed abnormal response patterns in aMCI, with reduced response to faces in MCI as compared to scrambled faces in right FFA/OFA [19]. Nonetheless, and contrary to previous studies, we used a face perception task that required dorsoventral integration, possibly explaining group differences. Indeed, an interesting association between sensitivity to stimulus depth and FFA’s response was found specifically for the MCI group, suggesting that the struggle to integrate dorsal and ventral information in MCI patients contributed to an increase sensitivity to lower level stimulus features, and consequently leading to the paradoxically higher response found for the scrambled faces. In the current study, we were able to investigate ventral visual stream functions in a wide range of localized object recognition regions, in well identified AD patients since all patients had clinical, cognitive, imaging, and/or CSF biomarkers. Other negative findings from our study are also in accordance with some behavioral studies. For instance, regarding the processing of objects (LOC), Revonsuo and colleagues [14] found that in early AD simple object recognition performance is still preserved, in contrast with more complex object categorization. In another study, AD’s accuracy in an object detection task was also normal when more time to respond was given to the participants [61].

The group differences identified with our approach, based on random effects analyses, provide support for the idea of a subtle, but significant, distinct functional response profiles of higher order visual processing areas in AD. Our results are also consistent with the observation of a 2-fold increase of neurofibrillary tangles and neuritic plaques in visual association cortex (BA18), compared to primary visual cortex (BA17), which is even more evident in higher-level visual processing areas [5, 62] including regions in the fusiform gyrus (∼BA 37) [63]. An early study by Giannakopoulos et al. [63] suggested that the presence of neurofibrillary tangles in fusiform gyrus/BA37 was significantly correlated with associative agnosia, where the difficulty relies on linking visual percepts to semantic memory [64]. Nevertheless, according to the work of Crutch et al. [65] visual agnosia (in both apperceptive and associative forms) are rare in mild stages of AD. Early deficits in visuoperceptive/visual recognition functions are more present in AD variants, such as posterior cortical atrophy [65–67]. Our AD sample was composed by patients with typical AD presentation at a mild stage of the disease, which might explain the subtle, but significant, effects found in response patterns in ventral higher-order visual processing areas.

The fact that the control group had higher responses on both the right FBA and the left VWFA compared with the AD group is consistent with previous evidence in mild stages of AD of both word, pseudoword and nonword reading impairments [68–72]. A few studies found abnormal word-reading scores in AD [68, 71] with further evidence of a correlation between word-reading score and the severity of cognitive impairment [68]. In another early study significant errors were found in pseudoword recognition in this disease, which the authors suggested to reflect an impairment in the use of more controlled processes of phoneme-grapheme decoding [69]. The presence of reading impairment in mild stages of AD can also be supported by one structural neuroimaging study that found a significant reduction in grey matter volume in left fusiform gyrus/left VWFA in both mild and moderate AD [73]. Authors suggested that this anatomical evidence could explain the reading deficits found in this disease.

In turn, and similarly to the VWFA, there is no previous neuroimaging evidence investigating functional impairment of the FBA in AD. There is support in the behavioral literature which may elucidate why AD patients had a lower response for their right FBA compared to controls. Previous findings pointed indeed to a diminished accuracy by AD patients in tasks containing the processing of silhouettes [12, 74, 75]. The need to access to relatively intact structural descriptions in semantic memory may partially explain this difficulty [74]. In fact, in our paradigm one of the bodies subcategories were body shape silhouettes. Furthermore, it has been suggested that AD patients are more susceptible to incomplete perceptual information in order to recognize objects, being impaired in processing degraded figures [10, 74, 76]. The presence of items requiring perceptual completion of a body could lead a diminished response by the FBA. Lastly, behavioral results of this study showed that AD patients had a worse performance compared to controls (<dprime;>omissions) for all stimuli. Since we designed a 1-back task to keep attention constant, it required intact object recognition and executive/attentional control, which is known to be impaired in AD patients, especially in what concerns processing speed [77–79] and sustained attention [80, 81]. In spite of this potential limitation, we controlled our analysis for performance differences, and the observed effects concerning the imaging results still hold true.

Given the executive task demands we also explored group differences in BOLD response to the most difficult stimulus found for both groups, that is, the scrambled material. This analysis corroborates the evidence that for the patients the scrambled material imposed even more demands than for the control group, since the former differentially recruited regions implicated in cognitive effort such as the prefrontal cortex (∼BA10), PCC, and Insula. Previous evidence implicating both the DLPFC in cognitive effort and executive demands has consistently been shown [49, 82–85]. The activation of the PCC is very interesting since this region is known to be an early target for the accumulation of Aβ plaques [86–88]. Also PCC is an area with complex connections with several brain networks being a region that takes part of episodic memory and the default mode network (DMN) [86, 87, 89]. The evidence showing that the frontal and cingulate regions are activated into a larger extent in AD is in congruence with the suggestion that in mild AD compensatory brain activity patterns may be present in regions involved in executive functioning and episodic memory [90, 91]. In fact, one study found evidence for increased prefrontal activation in AD patients in response to different cognitive tasks (semantic/recognition and face working memory tasks) suggesting that the recruitment of these regions might reflect a general compensatory effect in order to deal with task difficulty. Evidence of correlations between the activation of prefrontal regions, like the DLPC and task performance supports the idea of a compensatory prefrontal activation in AD patients [90]. The idea that the differential recruitment of prefrontal regions in AD might compensate for the loss of general cognitive resources is also corroborated by another study, where the activation of these regions was associated with a semantic memory task, contrary to controls, who recruited the same regions in more difficult episodic working memory task [91]. General decrease in deactivation in DMN [92], as well as a higher functional connectivity between those regions and fronto-parietal cortices have been discussed within the scope of a compensatory-recruitment hypothesis [93].

The present study has some limitations. The sample size is relatively modest. Moreover, the nature of functional disturbances in FBA and VWFA should be further investigated in future studies. Moreover, it remains to be understood how recognition requiring integration across ventral and dorsal stream pathways evolves in the natural history of AD.

This study provides a new perspective on how higher order visual recognition is affected early on in AD through the identification of a significant regional group difference in distinct regions of the ventral visual pathway with known hemispheric asymmetry. Knowing whether and how ventral visual pathway is affected in AD might be clinically relevant, especially in the context of visual disturbances that may occur in AD such as visual hallucinations.

## Supplementary Material

Supplementary Material

## Data Availability

The data supporting the findings of this study are available within the article and/or its supplementary material.
