# Supplementary material for: Hemispheric Patterns of Recruitment of Object Processing Regions in Early Alzheimer’s Disease: A Study Along the Entire Ventral Stream
Source: J Alzheimers Dis. 2023 Jan 31;91(3):1151–64. doi: 10.3233/JAD-220055 (PMC9912740; doi:10.3233/JAD-220055)
Supplement: Supplementary Material [file jad-91-jad220055-s001.pdf]

Supplementary Material

Hemispheric Patterns of Recruitment of Object Processing Regions in Early Alzheimer's Disease: A Study Along the Entire Ventral Stream

Supplementary Table 1. Mean BOLD signal changes for each category of stimulus in each ROI. Signal change for each condition was calculated by considering the time points of all baseline blocks in both runs.

Region		Alzheimer's (m; sd)	Controls (m; sd)
	Objects	0.56 (0.24)	0.55 (0.18)
	Scrambled	0.21 (0.12)	0.26 (0.10)
**R FFA**	Faces	**0.83 (0.40)**	**0.89 (0.42)**
	Bodies	0.74 (0.30)	0.75 (0.38)
	Places	0.47 (0.25)	0.40 (0.17)
	Verbal	0.57 (0.23)	0.31 (0.13)
	Objects	0.32 (0.15)	0.43 (0.16)
	Scrambled	-0.04 (0,09)	0,07 (0.04)
**L FFA**	Faces	**0.61 (0.38)**	**0.77 (0.30)**
	Bodies	0.36 (0.27)	0.72 (0.33)
	Places	0.23 (0.15)	0.31 (0.08)
	Verbal	0.14 (0.08)	0.43 (0.15)
	Objects	-0.01 (0.04)	-0.11 (0.09)
	Scrambled	-0.11 (0.11)	-0.23 (0.09)
**R STS**	Faces	**0.20 (0.14)**	**0.14 (0.08)**
	Bodies	0.02 (0.14)	0.16 (0.07)
	Places	-0.00 (0.06)	-0.14 (0.09)
	Verbal	0.08 (0.08)	-0.06 (0.06)
	Objects	0.12 (0.04)	-0.12 (0.07)
	Scrambled	-0.06 (0.07)	-0.17 (0.09)
**L STS**	Faces	**0.07 (0.05)**	**-0.05 (0.03)**
	Bodies	0.24 (0.06)	0.07 (0.08)
	Places	-0.01 (0.05)	-0.15 (0.09)
	Verbal	0.10 (0.04)	-0.04 (0.08)
	Objects	0.22 (0.08)	0.45 (0.15)
	Scrambled	-0.07 (0.09)	0.22 (0.07)
**R FBA**	Faces	0.42 (0.28)	0.78 (0.37)
	Bodies	**0.33 (0.18)**	**0.70 (0.34)**
	Places	0.13 (0.09)	0.28 (0.11)
	Verbal	0.11 (0.07)	0.29 (0.08)
	Objects	0.56 (0.24)	0.33 (0.14)
	Scrambled	0.21 (0.12)	0.12 (0.05)
**L FBA**	Faces	0.83 (0.40)	0.42 (0.17)
	Bodies	**0.74 (0.30)**	**0.53 (0.25)**
	Places	0.47 (0.25)	0.25 (0.07)
	Verbal	0.57 (0.24)	0.35 (0.13)

**R EBA**	Objects	0.44 (0.25)	0.41 (0.14)
	Scrambled	0.09 (0.09)	-0.01 (0.09)
	Faces	0.60 (0.32)	0.50 (0.35)
	Bodies	**0.88 (0.38)**	**0.95 (0.50)**
	Places	0.46 (0.23)	0.35 (0.17)
	Verbal	0.49 (0.13)	0.34 (0.15)
**L EBA**	Objects	0.28 (0.08)	0.13 (0.06)
	Scrambled	-0.11 (0.07)	-0.09 (0.05)
	Faces	0.17 (0.09)	0.18 (0.13)
	Bodies	**0.47 (0.18)**	**0.49 (0.23)**
	Places	0.15 (0.10)	0.08 (0.05)
	Verbal	0.21 (0.04)	0.12 (0.08)
**R PPA**	Objects	0.34 (0.11)	0.44 (0.13)
	Scrambled	0.21 (0.09)	0.31 (0.13)
	Faces	0.03 (0.07)	0.18 (0.06)
	Bodies	0.01 (0.07)	0.20 (0.06)
	Places	**0.67 (0.26)**	**0.79 (0.29)**
	Verbal	0.12 (0.08)	0.11 (0.05)
**L PPA**	Objects	0.30 (0.09)	0.29 (0.10)
	Scrambled	0.22 (0.06)	0.11 (0.09)
	Faces	0.15 (0.04)	0.11 (0.05)
	Bodies	0.09 (0.10)	0.10 (0.05)
	Places	**0.59 (0.24)**	**0.60 (0.24)**
	Verbal	0.17 (0.08)	0.03 (0.07)
**R LOCv**	Objects	**0.83 (0.36)**	**0.83 (0.38)**
	Scrambled	0.21 (0.11)	0.28 (0.27)
	Faces	0.76 (0.37)	0.84 (0.49)
	Bodies	0.82 (0.39)	1.01 (0.57)
	Places	0.71 (0.32)	0.66 (0.31)
	Verbal	0.55 (0.20)	0.53 (0.26)
**L LOCv**	Objects	**0.94 (0.38)**	**0.81 (0.38)**
	Scrambled	0.24 (0.17)	0.40 (0.26)
	Faces	0.82 (0.41)	0.64 (0.35)
	Bodies	0.94 (0.39)	0.87 (0.41)
	Places	0.73 (0.36)	0.77 (0.30)
	Verbal	0.75 (0.34)	0.60 (0.28)
**R VWFA**	Objects	0.22 (0.10)	0.52 (0.16)
	Scrambled	-0.05 (0.10)	0.25 (0.11)
	Faces	0.42 (0.29)	0.86 (0.39)
	Bodies	0.24 (0.22)	0.74 (0.36)
	Places	0.10 (0.11)	0.35 (0.16)
	Verbal	**0.05 (0.06)**	**0.31 (0.12)**
**L VWFA**	Objects	0.46 (0.17)	0.39 (0.15)
	Scrambled	0.17 (0.08)	0.05 (0.04)
	Faces	0.71 (0.32)	0.63 (0.24)
	Bodies	0.63 (0.25)	0.65 (0.29)
	Places	0.36 (0.19)	0.28 (0.07)
	Verbal	**0.49 (0.20)**	**0.41 (0.13)**

Supplementary Table 2. Between group post-hocs for the estimated effects in each ROIs. Table depicts the analysis using the beta values taken from ROIs' preferred category. Bold depicts the statistical results taken from right FBA and left VWFA. Statistical significance not corrected for multiple comparisons.

ROI	Contrast	Hemisphere	Alzheimer's versus Controls
FFA	[faces > places] AND [faces > scrambled] AND [faces > objects]	R	t (36) = -1.240, p > 0.222
		L	t (36) = 0.547, p > 0.587
STS		R	t (36) = 0.129, p > 0.897
		L	t (36) = 0.671, p > 0.506
FBA	[bodies > objects]	R	**t (29.865) = -2.957, p < 0.007**
		L	t (36) = -1.461, p > 0.152
EBA		R	t (36) = -0.811, p > 0.422
		L	t (36) = -0.403, p > 0.688
LOCv	[bodies > scrambled]	R	t (36) = -0.206, p > 0.837
		L	t (25.077) = 0.475, p > 0.638
PPA	[places > faces] AND [places > objects]	R	t (36) = -1.370, p > 0.178
		L	t (36) = -0.855 p > 0.397
VWFA	[verbal > scrambled]	R	t (36) = 0.968, p > 0.339
		L	**t (36) = -2.918, p < 0.007**
